# Women’s experiences of heavy menstrual bleeding and medical treatment: a qualitative study in primary care

**DOI:** 10.3399/BJGP.2022.0460

**Published:** 2023-03

**Authors:** Brittany Dutton, Joe Kai

**Affiliations:** Centre for Academic Primary Care, School of Medicine, University of Nottingham, Nottingham.; Centre for Academic Primary Care, School of Medicine, University of Nottingham, Nottingham.

**Keywords:** heavy menstrual bleeding, menorrhagia, primary healthcare, qualitative research

## Abstract

**Background:**

Heavy menstrual bleeding (HMB) is common and can affect women’s lives. Evidence on women’s experiences and their treatment of this problem after seeking primary care is lacking.

**Aim:**

To explore women’s experiences of HMB and their medical treatment up to 10 years after initial management in general practice.

**Design and setting:**

This was a qualitative study in UK primary care.

**Method:**

Semistructured interviews with a purposeful sample of 36 women who had participated in the ECLIPSE trial of medical treatments for HMB in primary care (levonorgestrel-releasing intrauterine system or other usual medical treatments — oral tranexamic acid, mefenamic acid, combined oestrogen–progestogen; or progesterone alone). Data were analysed thematically and a process of respondent validation was undertaken.

**Results:**

Women reported the wide-ranging and debilitating impact of HMB on their lives. They had often normalised their experience underlining persisting societal taboos about menstruation and reflecting low general awareness of HMB as treatable. Women commonly delayed seeking help for several years. They could then be frustrated by lack of a medical explanation for HMB. Women who had pathology identified felt able to make better sense of their HMB. Experiences of medical treatments varied considerably but were strongly influenced by the perceived quality of healthcare interactions with clinicians. Other influences on women’s treatment included considerations for their fertility, health concerns, family and peers, and views when approaching menopause.

**Conclusion:**

Clinicians should be aware of the considerable challenges faced by women with HMB; widely differing experiences of, and influences on, their treatment; and the value of patient-centred communication in this context.

## INTRODUCTION

Heavy menstrual bleeding (HMB) has been reported by 27–36% of women in surveys^1^^,^^2^ and is a common reason for consultation in primary care.^3^ There has been increasing recognition HMB can have an impact on women’s physical, social, emotional, and material quality of life.^4^^,^^5^ When evaluating how to help, outcomes such as quality of life and patient satisfaction are now considered as helpful as objective measures of blood loss,^6^ and this is reflected in clinical care guidelines.^3^

Most research has focused on the effectiveness of interventions to reduce menstrual blood loss^3^ and more recently on reducing the impact of HMB on quality of life.^7^^,^^8^ Earlier qualitative evidence from the 1990s and early 2000s highlighted the reality of the problem for women^9^^,^^10^ and social pressure to conceal symptoms.^11^^,^^12^ However, there has been a lack of more recent exploration of women’s experiences of HMB and of its medical treatment, which may be used for several years until menopause.

The ECLIPSE (Effectiveness and cost-effectiveness of Levonorgestrel containing Intrauterine system in Primary care against Standard treatment for Menorrhagia) trial recruited women presenting to their GP self-reporting HMB occurring in ≥3 consecutive cycles and who wished to have and were clinically assessed as appropriate for medical treatments.^7^^,^^8^

In line with recommended clinical guidance, treatment was started without investigation if history and examination suggested low risk of uterine pathology (no intermenstrual or post-coital bleeding or examination suggestive of fibroids or other disorders); or started after exclusion of pathology (by investigation with ultrasound and/or endometrial biopsy/hysteroscopy) when suggested clinically by history and examination.^3^

Consenting women were randomised to either a levonorgestrel-releasing intrauterine system (LNG-IUS in the form of the Mirena coil) or other usual medical treatment (oral tranexamic acid, mefenamic acid, combined oestrogen–progestogen or progesterone alone; chosen as clinically appropriate by the GP and women). Women could subsequently swap or cease their allocated treatments and their mean age at presentation was 42 years (standard deviation 4.9). The trial closed in 2015, reporting at 2 and 5 years.^7^^,^^8^

The current qualitative study was conducted as part of further observational follow-up of women 10 years after their presentation with HMB^13^ in the trial to explore their experiences of HMB and their treatment over this time.

## METHOD

### Sampling

A purposeful sample was selected from women willing and consenting to be interviewed, who participated in the wider follow-up study.^13^ Sampling was informed by women’s responses on self-reported 10-year outcome questionnaires to select women with diverse social demographics, and use and experience of treatment.

**Table:** How this fits in

Heavy menstrual bleeding (HMB) is known to significantly affect women’s health and quality of life, with pressure to conceal symptoms. Recent evidence on women’s experiences of HMB and its treatment after seeking primary care is lacking. This research shows the debilitating impact of HMB on women, and the challenges they can still face, including overcoming taboo and low general awareness that treatment can help. Women had widely differing experiences of current medical treatments for HMB in general practice and emphasised how they valued patient-centred communication in helping them.

### Data generation and analysis

Semi-structured interviews were conducted (August 2019 to December 2020) by a female health service researcher, face-to-face in women’s homes or by telephone, as preferred by participants (solely by telephone during the COVID-19 pandemic restrictions). Interviews were audiorecorded and transcribed verbatim. Interviews encouraged participants to speak freely about their experiences and followed a broad topic prompt developed initially with the help of two study patient and public involvement (PPI) advisers, and refined after early interviews. Women’s experiences and reflections about their HMB were explored, and its impact and treatment over time (topic guide provided in Supplementary Information S1).

Coding of interview transcripts was aided by application of NVivo software (version 12), with the field researcher and a second clinical primary care researcher identifying themes from the data.^14^

Data generation and analysis were iterative with sampling of women and further data generation continuing until no new themes emerged, suggesting saturation. To check and further refine interpretation, all the participants were invited to review and comment on a summary of preliminary findings from the analysis in a process of respondent validation.^15^ Study PPI advisers commented on and helped refine the readability of this summary before its circulation to participants. Further details on respondent validation is provided in the Supplementary Information S2.

## RESULTS

### Purposeful sample

In total, 145 of 206 (70%) women who responded in the 10-year follow-up study returned consent for potential interview, enabling selection of a purposeful sample of 36 women with a range of ages, social diversity and educational background, and with varied treatment experiences in the preceding 10 years. Characteristics of these women are summarised in Table 1.

**Table 1.** Characteristics of purposeful sample of women interviewed

**Characteristic**	**Purposeful sample (*n* = 36)**
**Age, years**	
Range	41–61
Median	55
Mode	60
Mean	54

**Self-defined ethnicity, *n***	
White British/English	30
Black British/Caribbean	3
South Asian	2
Mixed White/African	1

**Highest formal educational attainment, *n***	
No qualifications	5
GCSE or equivalent	13
NVQ3/A level or equivalent	8
Undergraduate degree	6
Postgraduate/higher degree	4

**Index of Multiple Deprivation category^a^, *n***	
1–3	16
4–7	13
8–10	7

**Single medical treatment only^b^, *n***	14
Still using LNG-IUS	4
No longer using LNG-IUS	6
Single oral medical treatment, never had LNG-IUS	4

**Surgical intervention^c^, *n***	5
After using LNG-IUS	2
After other medical treatment, never had LNG-IUS	3

**Multiple^d^ medical treatments and surgical intervention, *n***	15
Still using LNG-IUS	4
No longer using LNG-IUS	10
Never had LNG-IUS	1

**No treatment for HMB used between 5 and 10 years, *n***	2

a *Index of Multiple Deprivation, where decile 1 = most deprived to decile 10 least deprived, derived from postcode.*

b *Experienced use of one type of medical treatment (oral medication or LNG-IUS) and no surgical intervention in 10 years.*

c *Experienced surgical intervention (endometrial ablation or hysterectomy or other procedure for example for fibroid removal).*

d *Experienced use* ≥*2 different medical treatments. HMB = heavy menstrual bleeding. LNG-IUS = levonorgestrel-releasing intrauterine system.*

The participants had a similar age range, Index of Multiple Deprivation, and broad ethnic distribution to all those interested in being interviewed (*n* = 145), and to all women completing the 10-year follow-up (*n* = 206) (see Supplementary Table S2). In the quotes below, N refers to the study participant number.

### Impact of heavy menstrual bleeding and its taboo aspect

Women had experienced a profound and debilitating impact from HMB, affecting multiple aspects of their lives. They described flooding and unpleasant release of clots, and precautions they would take to manage or conceal their blood loss. They highlighted the burden of needing large amounts of sanitary products, soiling of bed linen and clothing, and avoiding social events and activities when menstruating. Intimate relationships suffered, including lack of libido and prolonged bleeding preventing sexual activity.

Some women attributed the breakdown of their relationships to HMB:

*‘ Our sex life sort of dwindled because of all of this* [HMB] *… I think relationships were affected … it sort of did away with my libido really. ’*

(N123, 55 years, no longer experiencing HMB, tranexamic acid, then LNG-IUS)

HMB often took a toll on women’s emotional wellbeing, with anxiety, low mood, and lack of confidence. Effects on working lives could be far-reaching causing embarrassment and stigma, and feeling pressure to conceal their menstruation:

*‘ I was just mortified, it always made me really anxious. I would get very tearful, erm I think because I was just scared. ’*

(N181, 55 years, no longer experiencing HMB, mefenamic and tranexamic acid then LNG-IUS)

*‘ I couldn’t go to work some days because I was just flooding … I didn’t want to tell* [manager] *what the problem was … I started to think am I going to lose my job if I carry on like this, it really affected me. ’*

(N128, 55 years, no longer experiencing HMB, tranexamic acid)

Women had concerns about volume of blood loss with their HMB and its implications, experiencing iron-deficiency anaemia and related hair loss. This sometimes preceded recognition of their HMB as the cause. Women highlighted low awareness of HMB and it not being taken seriously because it related to menstruation:

*‘ My hair was coming out in clumps and then* [GP] *told me I have got it because you’re severely anaemic …’*

(N202, 52 years, no longer experiencing HMB, contraceptive pill followed by LNG-IUS [removed], then hysterectomy)

*‘ Because it is coming from that part of your body, nobody cares really do they? ’*

(N429, 48 years, no longer experiencing HMB, mefenamic acid, contraceptive pill, then LNG-IUS)

Respondents highlighted how women not talking about HMB could reduce awareness and recognition of it as a problem that might be treatable.

Most respondents had tended to normalise the impact of their HMB, perceiving that this problem happened to everyone and so to simply persevere:

*’ You think, oh it can’t be that bad, … I am sure it will get better, you know, oh really do I want to bother them with this?’*

(N056, 41 years, no longer experiencing HMB, tranexamic acid followed by LNG-IUS then endometrial ablation)

*‘ I just thought this is normal … I have just got to stick with it until the menopause* [laughing]*, it never really occurred to me to try and seek help. ’*

(N191, 60 years, no longer experiencing HMB, contraceptive pill then LNG-IUS)

Women recognised this perseverance occurred in the context of continuing stigma and taboo about menstruation and HMB, which was not openly spoken about, or publicly portrayed, contributing to wider lack of awareness and knowledge among women generally that it could be helped:

*‘ It is silly really because, you know, half the population of the world have a period and it is … I don’t know why it is such a taboo still.’*

(N012, 56 years, no longer experiencing HMB, LNG-IUS then hysterectomy)

Respondents felt this taboo and lack of awareness was changing, with more and older women in the workforce. They felt availability of more diverse sanitary products and advances in treatments for HMB would also help. Nevertheless, it had often taken several years of HMB affecting their lives, relationships, or work before seeking help from their GP:

*‘ When it started causing problems with going to work and I started talking to people, I suddenly realised well maybe I better go and sort this out. ’*

(N123, 55 years, no longer experiencing HMB, tranexamic acid then LNG-IUS)

*‘ It just got me down … but it was probably four or five years before I kind of did anything, you know, about it.’*

(N051, 60 years, no longer experiencing HMB, contraceptive pill followed by LNG-IUS then hysterectomy)

### Understanding and making sense of heavy menstrual bleeding

A common frustration for many women was the apparent lack of a medical or pathological explanation for their HMB. They pondered ‘why me?’

In line with usual guidance, women had initiation of treatments in the ECLIPSE trial without further investigation if uterine pathology was not suspected clinically, or following investigations to exclude this if so indicated by history and examination. Thus, almost half of women interviewed had no investigations before starting treatments or found this happened only after failure of first or second treatment attempts.

Over time, those women with continuing HMB who later had pathology identified, such as development of polyps and fibroids, were able to make better sense of their HMB and treatment:

*‘ I had no problem whatsoever after I had the fibroids removed … I don’t know whether they knew about my fibroids* [before] *and … kept doing other stuff* [treatment for HMB] *… but I just felt it was a slow procedure* [process] *and I think to myself they could have got me in sooner to have the operation …’*

(N036, 60 years, no longer experiencing HMB, contraceptive pill then tranexamic acid, followed by surgical removal of fibroids)

Women also referred to getting older and approaching menopause. This sometimes caused delay in seeking help for HMB as they anticipated menopause itself. Other women attributed their HMB to starting after childbirth or to having a familial cause:

*‗ I have always had heavy periods but after the birth of my … child it got a lot worse. ’*

(N123, 55 years, no longer experiencing HMB, tranexamic acid, then LNG-IUS)

*‘ My daughter and granddaughter are going through it* [HMB] *now, it must be a family thing because my mum had an emergency hysterectomy at 44 because she was haemorrhaging … and I sort of started around the same time. ’*

(N214, 56 years, no longer experiencing HMB, norethisterone, then LNG-IUS contraindicated, then endometrial ablation)

### Quality of interaction with healthcare professionals

Women most consistently had positive experiences of their treatment at presentation or subsequently, when they trusted their GP or gynaecologist and communication in these encounters was perceived to be good. A positive experience occurred, even if multiple treatments were tried, when women felt fully informed about all their options and realistic expectations were set about the likelihood of success for each.

Women’s accounts underlined the value of joint decision making in discussing what may work best for them as individuals:

*‘ I felt he* [GP] *included me in any decisions … and he sort of didn’t say “this is what you must do ”, he said “how about if we try this and see how you get on? ” … Yes, trusted him 100%. ’*

(N016, 55 years, no longer experiencing HMB, contraceptive pill, followed by LNG-IUS, then hysterectomy)

*‘They would say* [name of GP] *“we don’t know if it will work you know but it is an option, give it a try ” … once you don’t trust your doctor it is a bit well where do you go? ’*

(N181, 55 years, no longer experiencing HMB, mefenamic and tranexamic acid, then LNG-IUS)

In contrast, those with negative experiences of their treatments had healthcare contact characterised by less communication or information sharing. Women could be concerned they were not being taken seriously or felt their HMB may not be considered a legitimate problem or recognised for its emotional impact.

Some women then felt denied the treatment they may have preferred. These women did not feel they had a say in treatment decisions or had felt less informed about their choices at different stages:

*’ *[Doctors] *just kept trying to say “it is nothing” and just kind of fobbing me off … it is almost as if they don’t understand the gravity of it … But it affects your life, doesn’t it?’*

(N301, 45 years, still experiencing HMB, contraceptive pill, followed by LNG-IUS)

*‘ I knew why I was having it* [treatment] *because of the bleeding … but I didn’t realise the overall effects of it, … none of that was explained … I don’t know what* [other] *options there were. ’*

(N012, 56 years, no longer experiencing HMB, LNG-IUS then hysterectomy)

Some questioned if their GP did not refer them to secondary care because of concerns about cost. They reported having to push for something to be done when their HMB was not improving:

*‘ I felt sometimes they used to just say “Oh have these tablets and you will be all right ” but I wasn’t — and I did go back but I don’t like to be a nuisance … ’*

(N004, 55 years, no longer experiencing HMB, tranexamic acid)

*‘ I did look around* [information] *and I had asked … would I be able to have that* [operation]*? And it was always … “loads of women have fibroids you just have to kind of put up with it .”’*

(N307, 44 years, no longer experiencing HMB, tranexamic acid, subsequent fibroids identified, hysteroscopic procedure, declined LNG-IUS, then hysterectomy)

Women advocated raising awareness of HMB. They suggested wider initiatives to get people talking about HMB, for example, with education in schools for boys and girls, in the workplace, and in the media.

### Influences on treatment choices and experiences of treatment use

Differing circumstances and considerations for women at different stages of their lives influenced their thinking about treatment options. Available choices for HMB in relation to fertility were important, either when younger and anticipating trying to start a family, or in retaining the future option to do so. Oral contraception or tranexamic acid could thus be continued for readily reversible or no contraceptive effect, respectively.

Other reasons for using oral medications included familiarity and ready control over their use (deciding when to use or stop them), even though effects on HMB might only have been partial. These factors could influence women’s decisions to avoid or delay using LNG-IUS when offered or considering endometrial ablation. For some women initiated on LNG-IUS at presentation, or switching to this having previously tried oral medical medications, the LNG-IUS had been transformative in reducing their HMB or causing amenorrhoea, albeit with advised delay in effect. They had gone on to have LNG-IUS replaced at 5 years and sometimes beyond at 10 years:

*‘ I had tried both of those* [mefenamic acid and tranexamic acid] *and it did control it* [HMB] *for a bit but didn’t really help a lot, …* [then] *I was actually not* [planning] *any more children … and my GP suggested the Mirena coil and … I have had one in* [5 years] *and* [then] *replaced … the Mirena has changed my life, absolutely, … it is not a quick fix … it took about six months for me* [HMB] *to settle down so I stuck with it. ’*

(N242, 50 years, no longer experiencing HMB, mefenamic and tranexamic acid, then LNG-IUS successful and used for over 10 years)

For other women, using LNG-IUS had been disappointing and unhelpful. They experienced greater unpredictability of their bleeding with LNG-IUS as practically unmanageable; or reported little effect on their HMB despite persisting up to a year or more, and so had had it removed. Sometimes this coincided with later discovery of other problems such as fibroids or polyps and subsequent surgical intervention:

*‘ *I did try it [progestogen-only pill] and … I started to bleed heavy again and back to the same old tiredness feeling … and I had the Mirena fitted, but it didn’t make any difference, it didn’t make any difference at all. *’*

(N228, 58 years, no longer experiencing HMB, contraceptive pill, followed by LNG-IUS then hysterectomy)

*‘ These clots came and I absolutely bled on to* [friend’s] *sofa and I was mortified … that was with the coil* [Mirena]*, I remember going back to the GP saying “this isn’t working ” … and then fibroids came up … so, then I had a hysterectomy …* [and] *was utterly relieved to be honest. It was a big, huge turnaround, it raised my quality of life infinitely. ’*

(N345, 58 years, no longer experiencing HMB, tablets ‘over the pharmacy counter’, then LNG-IUS, then fibroids identified and hysterectomy)

More invasive intervention by hysterectomy when other medical treatments had not helped was not deemed practically feasible or attractive for some women. However, the availability of less invasive endometrial ablation as an alternative had made a surgical option more possible:

*‘ They did give me the option of hysterectomy, but said I would be out of action for weeks and I thought how can I not drive the kids to school for weeks? ’*

(N019, 49 years, no longer experiencing HMB, mefenamic and tranexamic acid, followed by LNG-IUS, then endometrial ablation)

Women who had ceased taking their oral medical treatments over time had either experienced reduction or sometimes cessation of their HMB, or had switched to LNG-IUS. However, others had different or additional reasons for stopping treatments. These included individual health concerns, advice from family or peers, relationships finishing, or deciding to wait for the menopause. Health concerns included wanting to avoid hormone-based treatments, or women favouring surgery, to reduce future risks such as cancer:

*‘ I was originally on the pill to start with but because my mum and dad both had cancer that always made me very reluctant to continue anything hormone based. ’*

(N213, 55 years, no longer experiencing HMB, contraceptive pill, LNG-IUS, then fibroids removed)

*‘ There would be no site for ovarian cancer or anything like that, so I had the whole lot taken away. ’*

(N228, 58 years, no longer experiencing HMB, contraceptive pill, followed by LNG-IUS then hysterectomy)

*‘ I had spoken to my mum about it and she was like “no take it out* [Mirena]*, you shouldn’t have these things sort of stuck inside you ” … so I had it taken out. ’*

(N202, 52 years, no longer experiencing HMB, contraceptive pill followed by LNG-IUS [removed], then hysterectomy)

Approaching menopause, some women persevered with treatments despite these having less effect on their HMB than desired or opted for endometrial ablation after failure of oral treatments or LNG-IUS as they waited for natural menopause to occur.

Some reported retaining LNG-IUS beyond 10 years because they had not menstruated for several years and were afraid of their HMB returning or were uncertain if they had entered the menopause naturally:

*‘ I think at that point I realised my next step was a hysterectomy* [but] *… I kept thinking I was getting nearer what I thought would be the menopause. ’*

(N462, 59 years, no longer experiencing HMB, tranexamic acid followed by LNG-IUS, then opted for endometrial ablation)

*‘ I still kept with it* [Mirena] *because I just was so nervous about going back to my life* [with HMB] *… I didn’t know* [if] *I was kind of reaching the menopause … I was so frightened about going back to what I used to have. ’*

(N285, 54 years, no longer experiencing HMB, LNG-IUS since presentation and replaced at 5 and 10 years)

## DISCUSSION

### Summary

This study has found women’s experiences of HMB were debilitating with a wide-ranging impact on their lives, relationships, work, and wellbeing. Women had often normalised their experience of HMB, underlining wider persisting societal taboos about menstruation, and reflecting low general awareness of HMB as a treatable problem. Women could be affected by HMB for several years before they sought medical advice from their GP.

Women’s individual responses to treatments varied considerably, with failure or success from LNG-IUS or oral medical treatments over time. However, women’s experiences were consistently more positive when they felt their problem was acknowledged by clinicians, at presentation or at subsequent stages if their HMB was not improving. A positive experience occurred where there was a relationship of trust and women felt fully informed in discussing what may work best for them as individuals. A less successful or negative experience of treatment for HMB followed poor communication by professionals with women feeling unheard, dismissed or not taken seriously, and disempowered seeking effective treatment. Other influences on decisions about treatment over time included considerations for transition in womens’ lives, in relation to fertility, their health concerns, and when approaching menopause.

### Strengths and limitations

This study offers an exploration of women’s experience of HMB and its current treatment after presentation in primary care. Strengths include data generation with a purposeful sample that was socially diverse, engaging women with a range of differing experience. However, it is recognised these findings must be interpreted with regard to the selected sample as described. This sample was demographically and ethnically similar in range to women in the original trial and wider 10-year follow-up study.^13^ However, further research with women from diverse minority communities is needed as HMB experiences may differ, especially given the higher prevalence of fibroids in Black women.^16^

Women were interviewed by a female researcher, appropriate to the area of enquiry, and likely to have facilitated women fully sharing their experiences. Analysis of data was developed by two researchers of different disciplinary backgrounds. A process of validation with respondents themselves was also undertaken, confirming interpretation of their views and experiences as described.

### Comparison with existing literature

Women were as concerned with the wide-ranging effects of HMB on their physical and emotional health and quality of life as with HMB in itself. This is strongly consistent with earlier work.^4^^,^^9^^,^^17^^–^19 Similarly, menstrual concealment and taboo, women’s normalisation of HMB, and managing without seeking advice have been recurrent themes in previous studies,^11^^,^^20^^,^^21^ as found here. It is thus concerning the findings in the current study still echo other studies from previous decades, including about how women with HMB may feel dismissed by clinicians.^19^^–^23 Menstrual concealment and taboo contribute to low awareness and lack of open discussion about HMB in a reinforcing cycle^24^ with delay in or not seeking help.^2^ It is striking these issues remain for women in the 21st century despite advances in treatment.

The current research adds new insights about women’s experiences, including making sense of their HMB and influences on treatment, and why they may cease or continue using treatments. It illustrates women’s widely differing individual responses to currently available medical treatments for HMB. The study further underlines the challenges women still face and the essential importance of patient-centred care and communication in helping women. Parallel 10-year follow-up of women in the ECLIPSE trial cohort, with quantitative data on treatments women used, stopped or continued, is reported separately.^13^

Further exploration of factors influencing decision making about use of treatments or interventions for HMB might be undertaken, including direct observation of health consultations and exploration of the perspectives of GPs, primary care nurses, and gynaecologists.

### Implications for practice

Tackling the enduring taboo and stigma of menstruation and HMB remains a major challenge for improving women’s care. As the women interviewed here underlined, wider societal strategies for raising awareness are needed, alongside specific informational resources so women are aware that assessment and treatment of HMB can be helpful and are empowered to seek it. In this regard, primary care practitioners might more routinely ask women about their menstrual experience. They should be cognisant of the considerable challenges women with HMB face before seeking help overcoming taboo, normalising and tolerating HMB and its effects, and fear of not being taken seriously.

For clinical practice, the findings emphasise the importance and value to women of patient-centred communication in this context. Many of these messages have been highlighted in the new Women’s Health Strategy for England, which identifies menstrual health as a neglected priority area for improving women’s lives.^25^

In addition to managing HMB itself, health professionals should actively explore and acknowledge the wider impact of HMB on women’s lives. This might involve helping women feel listened to, empathy and support for anxiety or mood, or challenges in work or relationships. A clear explanation of HMB should be offered to achieve shared understanding with women that this is either considered benign HMB with no pathology suspected clinically^26^ or alternatively, HMB that may require investigation if history or examination suggests fibroids, polyps, or endometrial pathology (such as persistent intermenstrual bleeding) in line with recommended guidance for practice.^3^^,^^26^^,^^27^

Women will value good communication, use of appropriate information and shared decision making about treatments for their HMB tailored to their individual contexts, noting other influences for women and their changing needs or circumstances. For example, women may have differing expectations or preferences for management, depending on their age, requirements for contraception or fertility, or as they near the end of their menstruating lives.

Ongoing care should also ensure clinical willingness to continue to review women’s individual response to treatment as this can be expected to vary considerably, their need for further investigation, or different treatment or surgical options over time. This approach is likely to positively affect the quality of women’s care experience and satisfaction with treatment.
